# Skull base plasmacytoma in young patients aged below 40 years: Radiological perspectives and clinical outcomes

**DOI:** 10.1002/cnr2.2106

**Published:** 2024-07-05

**Authors:** Hesham Elsabah, Rola Ghasoub, Dina S. Soliman, Feryal Ibrahim, Mahmood B. Aldapt, Ruba Y. Taha, Safaa Al Azawi, Deena Mudawi, Abbas Moustafa, Halima Elomri, Honar Cherif

**Affiliations:** ^1^ Division of Hematology, Department of Medical Oncology National Center for Cancer Care & Research (NCCCR), Hamad Medical Corporation (HMC) Doha Qatar; ^2^ Department of Pharmacy National Center for Cancer Care & Research (NCCCR), Hamad Medical Corporation (HMC) Doha Qatar; ^3^ Department of Laboratory Medicine and Pathology National Center for Cancer Care & Research (NCCCR), Hamad Medical Corporation (HMC) Doha Qatar; ^4^ Department of Laboratory Medicine and Pathology Weill Cornell Medical College (WCMC‐Q) Doha Qatar; ^5^ Department of Clinical Pathology National Cancer Institute Cairo Egypt; ^6^ Internal Medicine Unity hospital, Rochester Regional Health Rochester New York USA; ^7^ Department of Radiology National Center for Cancer Care & Research (NCCCR), Hamad Medical Corporation (HMC) Doha Qatar; ^8^ Faculty of Medicine College of Medicine, Qatar University Doha Qatar

**Keywords:** autologous stem cell transplant, clivus, multiple myeloma, plasmacytoma, radiotherapy, skull base

## Abstract

**Background:**

Plasmacytoma of the skull base is a rare manifestation of plasma cell neoplasm with only a few cases documented in literature involving young adults. Plasmacytoma can be an isolated solitary lesion or a secondary manifestation of multiple myeloma (MM). In this study, we report the clinical and radiological characteristics, management, and outcomes of patients under the age of 40 who presented with skull base plasmacytoma and associated neurological manifestations. Additionally, we share our experience in treating a rare case of skull base plasmacytoma diagnosed during pregnancy, in which the patient exhibited a favorable response to myeloma treatment initiated after delivery.

**Case Series:**

Four patients were identified, comprising one pregnant female and three male patients, with a median age of 36 years (range 33–37 years). The main presenting symptoms were headache, dizziness, and cranial nerve palsy. All patients received underwent systemic myeloma therapy and radiotherapy with three patients also underwent autologous stem cell transplantation (ASCT). Notably, all patients achieved complete remission.

**Conclusion:**

Skull base plasmacytoma represents a rare manifestation of plasma cell neoplasms, underscoring the importance of considering it in the differential diagnosis of skull base lesions to ensure early intervention and avoid potential serious complications. Throughout our series, the cornerstone of therapy involved radiotherapy, systemic myeloma therapy, and ASCT, all of which elicited a favorable response in every case.

## 1 INTRODUCTION

Plasmacytoma is a solitary mass arising from the localized proliferation of neoplastic monoclonal plasma cells.^1^ Diagnosing this condition involves identifying clonal plasma cells in a tissue biopsy, along with a normal skeletal survey and no evidence of bone marrow involvement by clonal plasma cells or end‐organ damage.^2^ When bone marrow involvement by clonal plasma cells is 10% or greater, the diagnosis of multiple myeloma (MM) is typically given.^2^ Plasmacytoma is an uncommon tumor representing less than 3%–5% of plasma cell neoplasms^1^, ^3^ and is rarely described in patients under 40 years old.^4^ There are two distinctive forms of plasmacytoma distinguishable by the sites of origin: solitary bone plasmacytoma (SBP) (with vertebra and skull being the most affected sites) and extramedullary plasmacytoma (EMP) commonly originating from the head and neck regions, nasal and nasopharynx.^3^, ^4^, ^5^

Skull‐base plasmacytoma is an exceptionally rare clinical condition, with very few cases documented in the literature.^5^, ^6^, ^7^ Its occurrence in patients under the age of 40 is even more uncommon. Presenting symptoms vary depending on the extent of disease involvement, with cranial nerve damage being a common manifestation.^6^, ^7^, ^8^, ^9^

Radiotherapy serves as the primary treatment for isolated plasmacytoma. However, patients with MM typically necessitate systemic chemotherapy, which may include autologous stem cell transplantation (ASCT) for eligible patients.^2^

### 1.1 Patients and methods

Medical records of patients with plasma cell neoplasms who initially presented with skull base plasmacytoma and concurrent MM at the National Center for Cancer Care & Research (NCCCR) in Doha, Qatar between 2010 and 2020 were reviewed.

### 1.2 Results

Four patients with skull base plasmacytoma and concurrent MM were identified, comprising one pregnant female and three male patients, with a median age of 36 years (range 33–37 years). Two patients were typed as IgG Kappa, one patient as IgA Lambda, and one had lambda‐restricted plasmacytoma.

### 1.3 Case presentation

The clinicopathologic, management and biochemical findings are presented in Tables 1 and 2.

**TABLE 1:** Clinicopathologic and management data of four cases with skull base plasmacytoma.

#	Gender	Age/years	Type	Radiotherapy	Induction Chemotherapy	Conditioning + ASCT	Maintenance	Outcome	Follow up
1	F	36	IgA Lambda	30Gy skull base	VCD	Melphalan + ASCT	Lenalidomide	CR	42 m
2	M	37	IgG Kappa	30Gy skull base	VCD	Melphalan +ASCT	Lenalidomide	CR	85 m
3	M	35	Plasmacytoma‐lambda restricted	52Gy skull base	VCD + IT MTX	Melphalan +ASCT	None	CR	92 m
4	M	33	IgG Kappa	45Gy frontal and occiput	PAD	None	None	CR	8 m

Abbreviations: Cr, complete remission; F, female, M, male; IT MTX, intrathecal methotrexate; PAD, bortezomib, liposomal doxorubicin, dexamethasone; VCD, bortezomib, cyclophosphamide, dexamethasone.

**TABLE 2:** Biochemical markers of four cases with skull base plasmacytoma.

#	Hb g/dL	Cr mm/L	TP g/L	ALB g/L	Ca mm/L	LDH U/L	Mg mg/L	IgG mg/dL	IgA mg/dL	M‐serum g/L	M‐urine g/L	FLCK mg/L	FLCL mg/L	K/L	BM PCs%
1	8.7	109	80	27	3.9	340	5.3	398	2260	33 g IgA Lambda	Positive IgA lambda	9.3	261	0.036	2
2	12	70	89	38	2.4	118	1.6	2500	157	26 g IgG Kappa	10.3 g/L	374	17	22	75
3	17	65	65	40	2.6	N/A	1.69	968	405	Negative	Negative	N/A	N/A	N/A	2
4	14	60	84	48	2.4	175	1.9	1670	112	8 g IgG Kappa	Positive IgG Kappa	324	18	17	24

Abbreviations: ALB, albumin; B2M, beta2microglobulin; BM PCs, bone marrow plasma cells; Ca, calcium; Cr, creatinine; FLCK, free light chain kappa; FLCL, free light chain lambda; Hb, hemoglobin; K/L, kappa/Lambda ratio; M, monoclonal; N/A, not available; TP, total protein.

## 2 CASE 1

**FIGURE 1:**
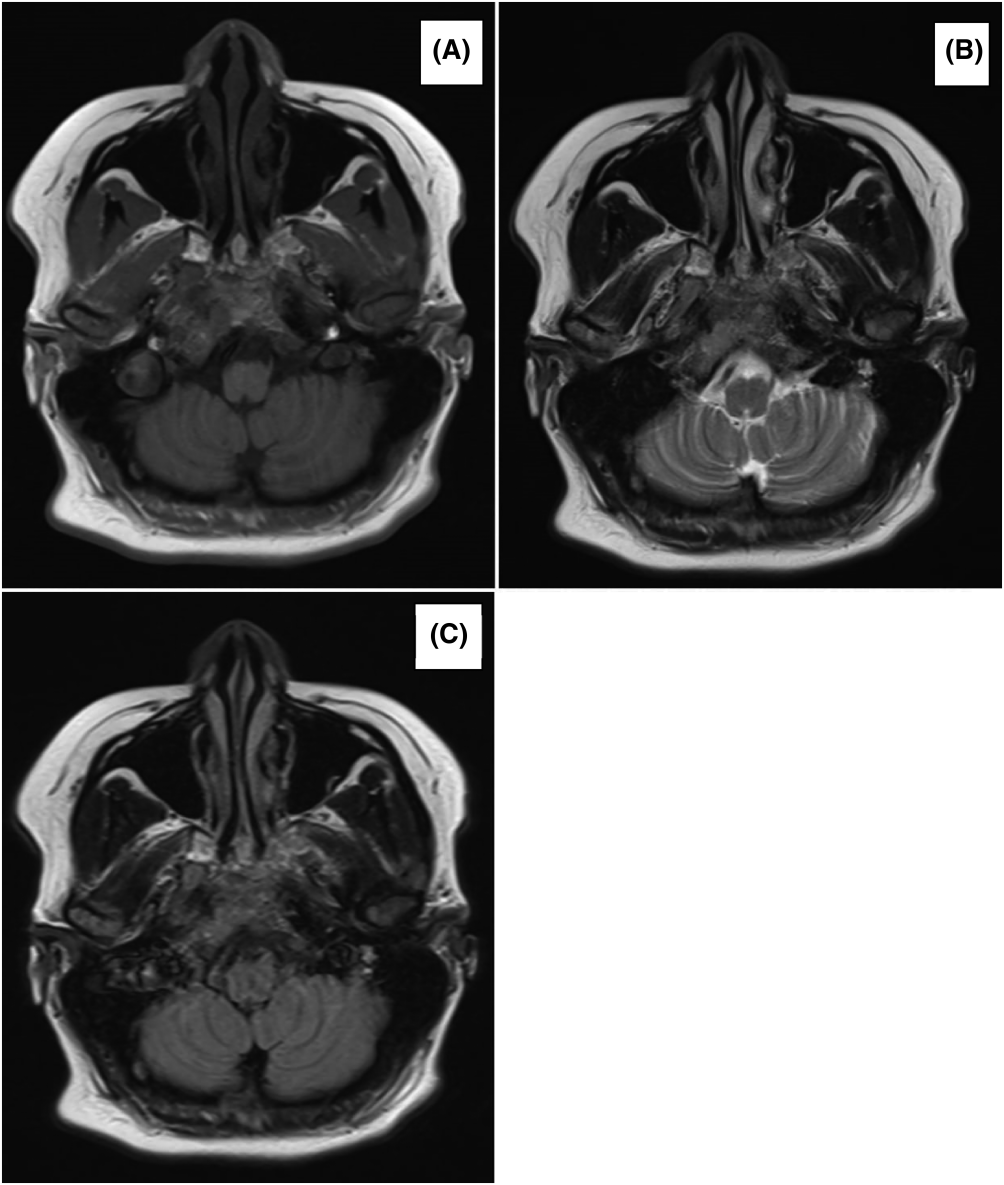
A magnetic resonance image (MRI) of the brain revealed marked heterogeneity of marrow signal, involving clivus and extends to petrocival bilaterally, sphenoid and abutting the cavernous sinus. (A) T1; (B) T2; (C) T1 post contrast.

A 36‐year‐old, 30‐weeks pregnant women (gravida 5, para 3) presented with diplopia persisting for 3 days. She denied any history of headache or blurred vision. Neurological examination revealed sixth cranial nerve palsy in the left eye. Due to severe intrauterine growth retardation and oligohydramnios, the patient underwent emergency caesarian section. Following the procedure, the patient gave birth to a baby with low birth weight (975 g). Magnetic resonance imaging (MRI) of the brain revealed marked heterogeneity of marrow signals, with a lesion involving the clivus and extending to the petroclival region bilaterally, sphenoid, and abutting the cavernous sinus (Figure 1). Biopsy of the lesion confirmed the diagnosis of plasmacytoma. Further work‐up revealed anemia with hemoglobin (Hb) of 8.7 g/dL (normal 12–16 g/dL), hypercalcemia with calcium of 3.9 mmol/L (normal 2.15–2.5), and a monoclonal band typed IgA lambda (33 g/L) was detected by serum protein electrophoresis (SPEP). BM aspirate showed 2% of clonal plasma cells. Computed tomography (CT) revealed multiple bony lytic lesions, confirming the diagnosis of MM. The patient underwent treatment abroad consisting of radiotherapy to the base of the skull (30 Gy) over 2 weeks and bortezomib‐cyclophosphamide and dexamethasone (VCD) chemotherapy for three cycles, followed by ASCT and lenalidomide maintenance. A complete clinical remission was achieved.

## 3 CASE 2

**FIGURE 2:**
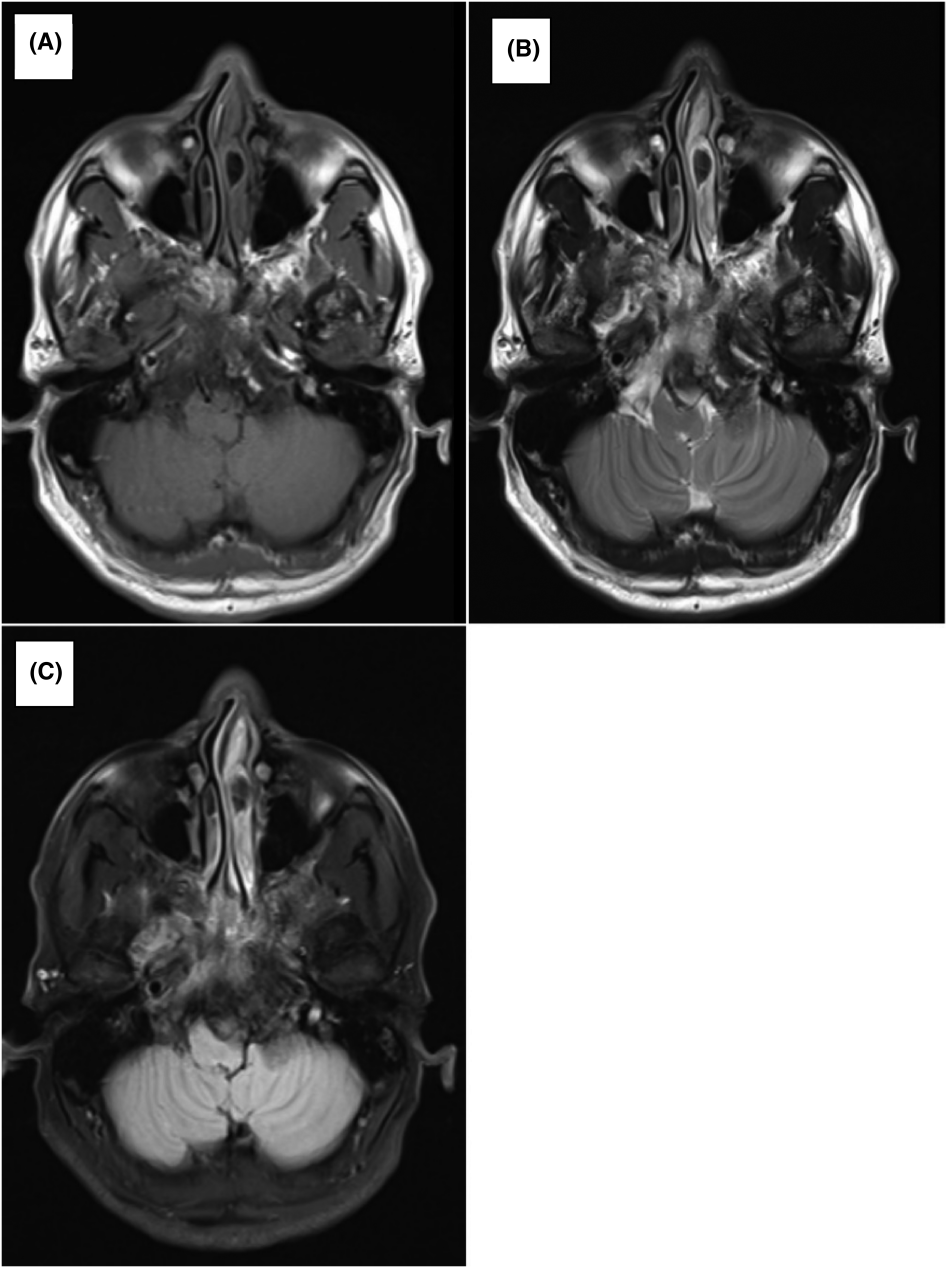
Magnetic resonance image (MRI) brain revealed a destructive skull base lesion eroding the body of sphenoid, clivus mainly inclined toward the right side down to the jugular fossa and occipital condyle with soft mass extending to prepontine, seller, parasellar, and right petrous bone. (A): T1 pre‐contrast; (B) T2 post‐contrast; (C) FLAIR.

A 37‐year‐old male presented with headache, dizziness, and tinnitus in the right ear. Neurological examination was unremarkable. MRI of the brain revealed a destructive skull base lesion eroding the body of the sphenoid, clivus down to the jugular fossa, and occipital condyle with soft mass extending to the prepontine, seller, parasellar and right petrous bone (Figure 2). Skull tissue biopsy confirmed the diagnosis of plasmacytoma. SPEP showed a monoclonal band typed as IgG kappa (26 g/L). BM biopsy showed extensive involvement by plasma cells. The patient underwent treatment with [3D conformal] radiotherapy to the base of the skull (30 Gy) in 10 fractions and VCD combination followed by ASCT and lenalidomide maintenance. He achieved complete remission.

## 4 CASE 3

**FIGURE 3:**
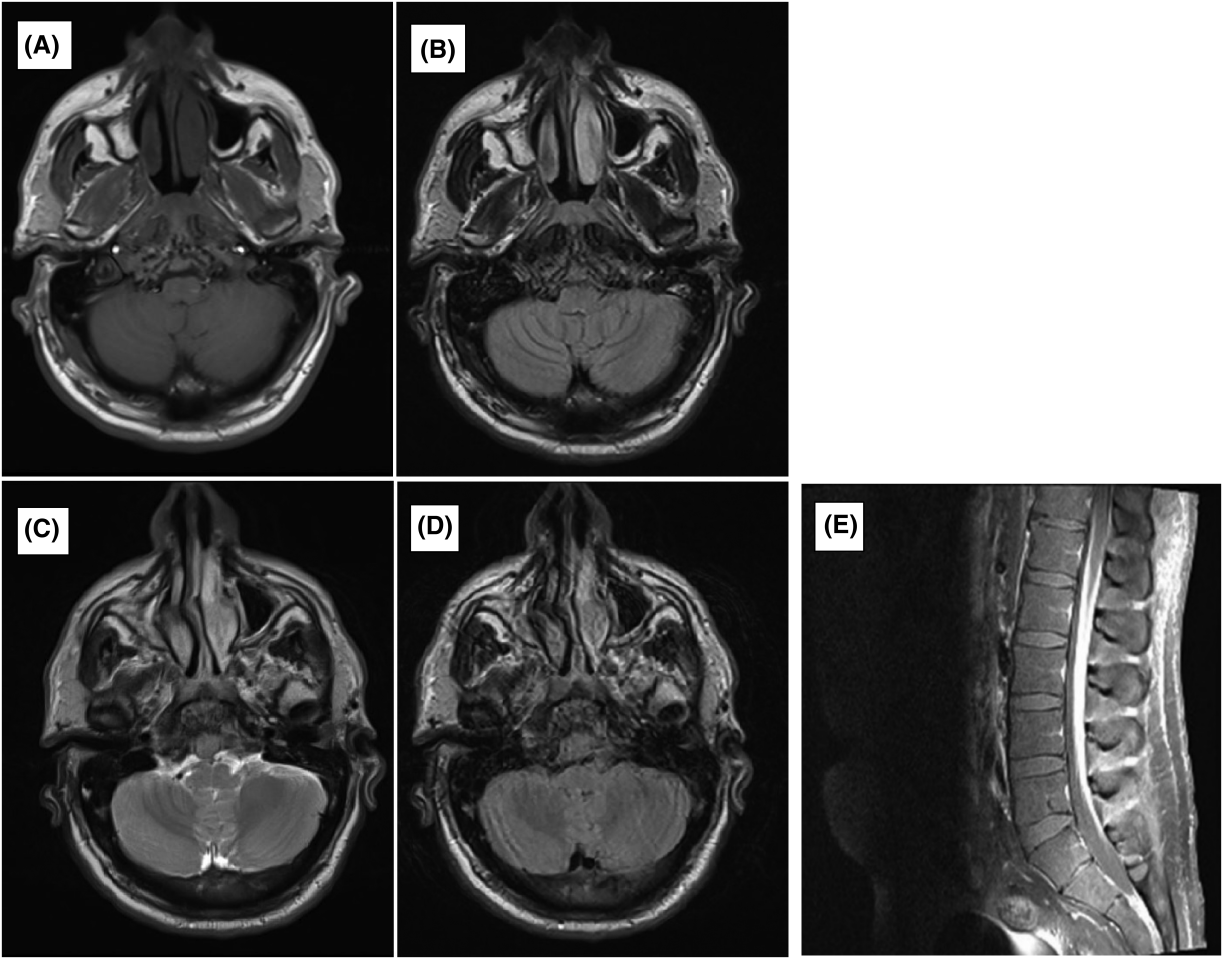
Magnetic resonance image (MRI) brain clivus mass lesion with soft mass in the prepontine cistern extending to the sphenoid and occipital condyle, involving both cavernous sinus tentorium of cerebelli and invading the pituitary gland (A: T1 pre‐contrast; B: FLAIR; C: T2, D: Post‐contrast). (E) Sagittal selected image of the lumbosacral spine (T1 post contrast) with appreciable increased enhancement along the cauda equina roots and surrounding the conus medullaris.

A 35‐year‐old male presented with chronic headache and diplopia for 5 days. Neurological examination revealed a left‐sided sixth cranial nerve palsy. MRI brain revealed clivus mass lesion with soft mass in the prepontine cistern extending to the sphenoid and occipital condyle, involving both cavernous sinus, tentorium of cerebelli, and invading the pituitary gland (Figure 3A–D). Biopsy from the lesion revealed a lambda light‐chain‐restricted plasmacytoma. Other relevant laboratory tests, BM aspirate and biopsy findings were normal, indicating solitary plasmacytoma.

The patient was treated abroad with radiotherapy to the skull base (52 Gy) in 27 fractions. Six weeks later, he presented with progressive lower limb weakness with no sensory level. MRI spine revealed extensive enhancement of the conus medullaris and intrathecal nerve root of the lumbar and sacral regions (Figure 3E). Cerebrospinal fluid (CSF) analysis revealed turbid fluid with white blood cell (WBC) count of 25 × 103/μL (69% lymphocytes, 28% monocytes, and 2% neutrophils), glucose 5.5 mmol/L and protein 3.4 g/L. CSF flow cytometry revealed 1% CD38/CD138 lambda‐restricted clonal plasma cells. The patient started on intrathecal methotrexate along with VCD combination for six cycles followed by ASCT, and he achieved complete remission.

## 5 CASE 4

A 33‐year‐old male presented with transient loss of consciousness. Physical examination results were unremarkable. MRI of the brain revealed multiple enhancing calvaria and skull base lesions involving the right aspect of frontal bone as well as left basiocciput. Skull base tissue biopsy revealed plasmacytoma, and BM biopsy confirmed the diagnosis of MM. The patient received intensity‐modulated radiotherapy [IMRT] to the left frontal region (45 GY) and left basiocciput (45 Gy) in 25 fractions each, followed by chemotherapy (bortezomib, liposomal doxorubicin, and dexamethasone). He achieved complete clinical remission.

## 6 DISCUSSION

We report the clinical presentation, radiological findings, and treatment outcomes of four patients presented with skull base plasmacytoma. Plasmacytoma is a rare malignancy, accounting for only 5% of all plasma cell neoplasms.^2^, ^3^ It is categorized into two types: SBP and EMP.^4^, ^5^ SBP has a high incidence and is associated with poor prognosis owing to its increased likelihood of progressing to MM.^5^ Anterior lesions in the nasopharyngeal region and central lesions in the clivus, sphenoid, and petrous apex are the most observed skull involvements.^6^, ^7^, ^8^ Skull base plasmacytoma is rare in younger adults (<40 years of age).^4^ Our patients in this series were all at the young age group (median 36 years) which is very uncommon for plasma cell malignancies. The age of our patients most probably reflects the much younger population in our society (median age 33 years) compared with Western countries (median age >40 years).^10^ The likelihood of solitary plasmacytoma progressing into MM or experiencing relapse within the first 3 years of diagnosis is relatively significant, with rates ranging from 14% to 38%.^11^

MM presented as skull base plasmacytoma is an exceedingly rare clinical entity, primarily documented in case reports within the existing literature.^6^, ^9^ In our series, the diagnosis of MM was established in three patients, while one patient had isolated plasmacytoma, that progressed shortly after radiotherapy with bilateral lower limb weakness and extensive involvement of conus medullaris. These findings suggest that skull base plasmacytomas may be indicative of a systemic plasma cell neoplasm rather than solitary plasmacytoma. Among the patients diagnosed with MM, two were classified as IgG Kappa subtype, one as IgA Lambda subtype, and the fourth patient had lambda‐restricted plasmacytoma. Unfortunately, cytogenetics characterization was not available.

Patients with skull base plasmacytoma present with a range of manifestations, including headache, cranial nerve palsies, and otalgia.^5^ In our case series, the patients presented with various neurological symptoms such as headache, diplopia, dizziness, tinnitus, and cranial nerve palsy, depending on the site of involvement. Anemia and hypercalcemia were observed in only one patient, and all patients had normal renal function. In most patients in this series, the tumor originated from the clivus. Two patients exhibited normal or minimal BM involvement by clonal plasma cells. The location of the disease likely contributes to early symptomatology and facilitates early diagnosis before the onset of systemic myeloma manifestations.

Plasma cell neoplasms diagnosed during pregnancy are rare with only 46 cases of MM during pregnancy documented in the literature.^12^, ^13^ Diagnosing MM in this context can be challenging, as myeloma is very uncommon in young females and presenting symptoms such as back pain and anemia can be easily overlooked and are commonly attributed to pregnancy. In addition, diagnostic radiological investigations are usually undesirable during pregnancy due to the potential risk of fetal exposure.^12^, ^13^, ^14^ Moreover, consistent guidelines for the management of this rare condition are lacking. However, favorable outcomes have been reported.^15^ To the best of our knowledge, this is the first case report of skull base plasmacytoma diagnosed during pregnancy. Our patient responded well to myeloma treatment initiated after delivery.

Local radiotherapy is the treatment of choice for isolated skull base plasmacytoma. The recommended radiation therapy dose according to established guidelines for the involved site is 40–50 Gy in 1.8–2.0 Gy fractions, with a total of 20–25 fractions.^11^

However, there is a scarcity and heterogeneity of data on skull base plasmacytoma and concurrent MM. Patients with MM require combination therapy including novel agents, such as proteasome inhibitors (PI) and immunomodulatory drugs (IMID) followed by ASCT for eligible patients.^16^, ^17^ A meta‐analysis of 47 patients revealed that 47% underwent surgical resection, 53% had radiation therapy, while 11 patients with concurrent MM received adjuvant therapy. The 2‐year and 5‐year overall survival rates were reported to be 78% and 59%, respectively.^5^

Regarding management, all our patients received induction therapy with a bortezomib‐based regimen and three patients underwent ASCT. Two patients received maintenance therapy with lenalidomide. Radiotherapy was given to all patients with doses ranging between 30 and 52 Gy. Post‐therapy evaluation revealed complete remission in all patients. This discrepancy in treatment reflects the evolving knowledge of MM management over the 10‐year period.

Our case series presents both strengths and limitations. In this case series, we report cases of a rare entity of skull base plasmacytoma manifesting as MM in young individuals, for which there is a scarcity of information in the literature. Additionally, we report a rare case of skull base plasmacytoma diagnosed during pregnancy. Despite the limited number of cases, our case series is significant because of the rarity of the disease, and which can make a valuable contribution to the current literature. Furthermore, we provide details of chemotherapy treatment and patient outcomes, which can assist clinicians in making treatment decisions for future cases.

## 7 CONCLUSION

Skull base plasmacytoma represents a rare manifestation of plasma cell neoplasms, underscoring the importance of considering it in the differential diagnosis of skull base lesions to ensure early intervention and avoid potential serious complications. Throughout our series, the cornerstone of therapy involved radiotherapy, systemic myeloma therapy, and ASCT, all of which elicited a favorable response in every case.

## AUTHOR CONTRIBUTIONS

**Rola Ghasoub:** Writing – review and editing. **Hesham Elsabah:** Validation; conceptualization; writing – original draft; writing – review and editing. **Dina S. Soliman:** Writing – review and editing. **Feryal Ibrahim:** Writing – review and editing. **Mahmood B. Aldapt:** Writing – review and editing. **Ruba Y. Taha:** Writing – review and editing. **Safaa Al Azawi:** Writing – review and editing. **Deena Mudawi:** Writing – review and editing. **Abbas Moustafa:** Methodology; writing – review and editing. **Halima Elomri:** Writing – review and editing. **Honar Cherif:** Writing – review and editing.

## CONFLICT OF INTEREST STATEMENT

The authors have stated explicitly that there are no conflicts of interest in connection with this article.

## ETHICS STATEMENT

The study was approved by Medical Research Center, Hamad Medical Corporation, under MRC number: MRC 04‐23‐800.

## PATIENT CONSENT STATEMENT

All patients have consented to the usage of their clinical data.

## Data Availability

Data sharing not applicable to this article as no datasets were generated or analysed during the current study.
